# Effect of Continuous Positive Airway Pressure Treatment on Hearing and Inner Ear Function in Patients with Obstructive Sleep Apnoea—Original Research

**DOI:** 10.3390/medicina61101833

**Published:** 2025-10-14

**Authors:** Mirjana Grebenar Čerkez, Željko Zubčić, Stjepan Jurić, Jelena Šarić Jurić, Jelena Kovačević, Željka Laksar Klarić, Darija Birtić

**Affiliations:** 1 Faculty of Medicine Osijek, Josip Juraj Strossmayer University of Osijek, 31000 Osijek, Croatia; mirjanagrebenar@gmail.com (M.G.Č.); zzubcic21@gmail.com (Ž.Z.); jelenasaricjuric@gmail.com (J.Š.J.); dr.kovacevic.jelena@gmail.com (J.K.); zeljka.l.klaric@gmail.com (Ž.L.K.); 2 Department of Otorhinolaryngology and Head and Neck Surgery, University Hospital Center Osijek, 31000 Osijek, Croatia; 3 Department of Neurology, University Hospital Center Osijek, 31000 Osijek, Croatia

**Keywords:** cochlear receptor cells, obstructive sleep apnoea, otoacoustic emission, pure-tone audiometry

## Abstract

*Background and Objectives*: This study aimed to investigate the influence of continuous positive airway pressure in patients with obstructive sleep apnoea on hearing and the possibility of recovering cochlear receptor cells. *Materials and Methods*: Forty-two patients with obstructive sleep apnoea (OSA) were assigned to the study group. Patients underwent pure-tone audiometry and transient-evoked (TEOAE) and distortion-product (DPOAE) otoacoustic emissions before starting continuous positive airway pressure (CPAP) therapy and six months after CPAP therapy. Subjects were further divided into the following two groups: those who adequately used the therapy and those who did not adhere to treatment recommendations. *Results*: There is no significant difference in hearing thresholds for specific frequencies after six months of CPAP therapy. There is no significant difference in TEOAE and DPOAE SNR values at any frequency after 6 months of CPAP therapy. There is no significant difference in hearing threshold results for specific frequencies as a function of subject co-operation with treatment. After therapy, there is a significant difference in the SNR values in TEOAEs at 2 kHz and 4 kHz in subjects of the OSA target group, depending on co-operation, being higher in co-operative subjects, while there are no significant differences at other frequencies. There is a significant difference in the SNR results in DPOAEs, where they are higher in co-operative subjects at 1000, 6000, 7000 and 8000 Hz. *Conclusions*: The use of continuous positive airway pressure as a therapy for OSA has no effect on hearing and cochlear receptor cell recovery. Co-operation with CPAP treatment does not affect hearing threshold, but does affect cochlear receptor cell function, which is better at mid and higher frequencies in those who co-operate. These findings underscore the clinical significance of treatment adherence. Consistent adherence is associated with measurable improvements in hearing, particularly at higher frequencies, which cannot typically be evaluated in routine clinical practice using standard pure-tone audiometry. Our results emphasise the importance of promoting compliance with CPAP therapy not only for cardiovascular and neurological protection, but also for maintaining hearing health.

## 1. Introduction

Obstructive Sleep Apnoea (OSA) is a sleep-related breathing disorder characterised by recurrent episodes of partial or complete upper airway obstruction during sleep lasting at least 10 s. This leads to either partial (hypopnoea) or complete (apnoea) cessation of breathing, resulting in hypoxia in the body’s cells [1,2]. The diagnosis is made by polysomnography, in which cardiovascular, neurophysiological and other signals are recorded during a complete night’s sleep [3]. The results are used to determine the apnoea/hypopnoea index (AHI), which classifies the condition as mild (5–15/h), moderate (15–30/h), or severe (˃30/h) OSA [1,4].

The first and most commonly used therapy method for patients is treatment with a device that generates continuous positive airway pressure, known as a continuous positive airway pressure (CPAP) device. CPAP is considered the gold standard for the treatment of moderate to severe OSA. The device uses tubing and a nasal or oronasal mask to deliver positively pressurised air into the upper airway and keep it open [1,5,6]. There are different types of PAP devices: CPAP (continuous positive airway pressure), BiPAP (bi-level positive airway pressure), and auto-titrating PAP devices (auto CPAP or APAP), which are used in the vast majority of cases today [5,6]. For the treatment to be considered effective, the patient must use the device for at least 4 h during night-time sleep on at least 70% of nights. CPAP adherence was assessed using the Centers for Medicare and Medicaid Services (CMS) definition, which considers patients adherent if the device is used for at least 4 h per night on at least 70% of monitored nights. Patient compliance with proper use of the device is around 70% [7,8].

The cochlea has a high antioxidant capacity [9]. The receptor cells are particularly sensitive to oxidative stress as they do not divide during life and thus accumulate oxidative damage. They are also highly specialised, relatively large and have a high metabolic activity to ensure an adequate energy supply [10,11]. Conditions that lead to reduced blood flow to the inner ear (e.g., ageing, hypoxia) cause excessive production of reactive oxygen species, leading to destruction of mitochondrial DNA and damage to inner ear structures [11].

For this reason, patients with OSA have a higher risk of sensorineural hearing loss compared to healthy individuals. Studies that have investigated the effects of obstructive sleep apnoea on hearing have shown that it involves damage to the outer hair cells of the cochlea [12,13]. However, it is unknown how continuous positive airway pressure therapy affects the inner ear and the function of the cochlear receptor cells.

The aim of this study was to investigate the effects of CPAP therapy in patients with obstructive sleep apnoea on hearing and the potential for restoring cochlear receptor cell function.

## 2. Materials and Methods

This study was designed as a prospective cohort study and was conducted at the University Hospital Center Osijek, Croatia, which provides healthcare services to approximately 650,000 inhabitants across five counties. The Department of Neurology performs approximately 200 initial polysomnographies, CPAP titrations, and follow-up polysomnographies annually. The study period extended from October 2021 to July 2024 and largely overlapped with the COVID-19 pandemic (officially declared over in May 2023). Before the study onset, a lockdown period had substantially reduced the provision of healthcare services, including the number of polysomnographies performed. In the post-COVID period, there was an increased influx of patients with a pre-existing diagnosis of obstructive sleep apnoea (OSA) requiring follow-up assessments, compared with newly diagnosed cases of moderate or severe OSA. Based on a power analysis (G*Power, version 3.1.9.2, Kiel University, Germany), a minimum sample size of 28 participants was required to detect large differences, assuming a significance level of 0.05, statistical power of 80%, and an effect size of d = 0.5. A total of 42 adult patients with newly diagnosed moderate to severe obstructive sleep apnoea, defined as an apnoea–hypopnoea index (AHI) ≥ 15 events per hour, were included in the study and prospectively followed. Inclusion criteria were: age between 18 and 69 years, a new diagnosis of moderate or severe OSA, and a clinical recommendation for CPAP therapy. Exclusion criteria included: conductive hearing loss, a history of neurotological disorders, sudden sensorineural hearing loss, professional noise exposure, type II diabetes mellitus and age under 18 or over 69 years. All participants were overweight or obese (BMI > 25 kg/m^2^) and had hypertension as a comorbidity. All participants started CPAP therapy and agreed to a 6-month follow-up. To assess treatment adherence, data were retrieved from the CPAP device memory cards. Patients who had not used the device at all—and therefore had no recorded data—also declined to participate in the study. The audiological examination included pure-tone audiometry and otoacoustic emissions testing—both transient-evoked (TEOAE) and distortion-product (DPOAE)—performed at baseline (before starting CPAP therapy) and at the 6-month follow-up. Pure-tone audiometry is a standardised, subjective method for assessing hearing thresholds in a specific frequency range. The tests are performed with a calibrated audiometer at octave and interoctave frequencies between 500 Hz and 8000 Hz. Air conduction thresholds are measured with supra-aural headphones, while bone conduction thresholds are measured with a bone vibrator placed on the mastoid process. Pure tones are presented in ascending intensity, and the lowest intensity level at which the subject consistently perceives the sound is recorded as the hearing threshold and provides information about the degree and type of hearing loss [14]. The otoacoustic emission (OAE) testing is an objective and non-invasive technique for assessing the functional status of the outer hair cells in the cochlea. In this test, a probe with a miniature loudspeaker and a sensitive microphone is inserted into the external auditory canal. Acoustic stimuli are delivered into the ear and the probe detects the sound energy reflected by the cochlea. The presence and amplitude of these emissions indicate normal function of the outer hair cell and the integrity of the auditory pathway. Transient-evoked otoacoustic emissions are triggered by short-click stimuli and provide an overview of global cochlear function, especially in the frequency range of 1000–4000 Hz [14,15]. Distortion-product otoacoustic emissions, on the other hand, are elicited by two continuous pure tones (f1 and f2) and provide frequency-specific information about cochlear function over a broader range, allowing for more detailed topographic assessment [14,15]. After the 6-month follow-up, participants were divided into two groups based on adherence to CPAP therapy: patients who adhered to the therapy and those who did not.

### Data Analysis

Categorical variables were summarised using absolute and relative frequencies. Ordinal and continuous variables were described using the mean, standard deviation, median, range, and interquartile range, as appropriate. Differences between two independent groups on ordinal outcomes were assessed using the Mann–Whitney U test, while the Wilcoxon signed-rank test was used for paired ordinal data. The normality of continuous variables was evaluated using the Shapiro–Wilk test. Following the Central Limit Theorem, samples exceeding 50 observations were assumed to approximate a normal distribution, regardless of the test outcome. For comparisons of means between two independent groups with normally distributed data and equal variances, Student’s *t*-test was applied. In cases of unequal variances, as indicated by Levene’s test, Welch’s *t*-test was used. For small independent samples not meeting the normality assumption, the nonparametric Mann–Whitney U test was employed. Although the dependent samples were sufficiently large, the distribution of the differences between paired measurements was formally tested with the Shapiro–Wilk test, as the paired-samples *t*-test lacks robustness to non-normality. A two-tailed *p*-value < 0.05 was considered indicative of statistical significance. All analyses were performed using IBM SPSS Statistics for Windows, Version 27.0 (IBM Corp., Armonk, NY, USA), MedCalc Statistical Software, Version 22.023 (MedCalc Software Ltd., Ostend, Belgium; https://www.medcalc.org; 2024), and TIBCO Statistica, Data Science Workbench, Version 14 (TIBCO Software Inc., Palo Alto, CA, USA; http://tibco.com).

## 3. Results

The age of the participants ranged from 29 to 69; the mean age was 52.43 years (Table 1).

Table 2 shows the distribution of patients according to the degree of apnoea into those with moderate and those with severe apnoea. In the study, 11 subjects had moderate OSA, and 31 had severe OSA.

All 42 participants underwent an otoscopic examination and tympanometry to rule out the presence of conductive hearing loss. Subsequently, bilateral pure-tone audiometry was performed both at the beginning (before starting CPAP therapy) and after a 6-month treatment period. Autotitrating positive airway pressure (Auto-CPAP) devices were used to treat obstructive sleep apnoea in all study participants.

The audiometric outcomes are summarised in Table 3.

In all analysed cases, the paired-samples *t*-test showed that the differences in pure-tone audiometry results for both ears, before and after CPAP therapy, were not statistically significant.

Following the audiometric examination, otoacoustic emission tests were performed in all participants using the two currently available diagnostic modalities: transient-evoked OAE and distortion-product. The main parameter analysed was the signal-to-noise ratio (SNR), where values above 6 dB are considered indicative of a normal cochlear response at the frequency tested [14]. Results are presented in Table 4 and Table 5.

TEOAE SNR values did not show statistically significant changes at any of the tested frequencies after 6 months of CPAP therapy. DPOAE SNR values did not show statistically significant changes at any of the tested frequencies after 6 months of CPAP therapy.

After six months of therapy, patients were categorised into two groups based on their adherence to therapy: adherence to therapy and non-adherence.

Table 6 shows the distribution of patients in the OSA group according to their adherence. The data were obtained from the SD memory card integrated into each CPAP device [5]. Approximately two-thirds of participants showed good adherence, while almost one-third were categorised as not adherent.

According to the CMS criteria, 69% of participants were classified as adherent to CPAP therapy. The mean nightly usage across the cohort was 5:44 h, and the device was used on 78.63% of monitored nights.

For all participants in the OSA group, the apnoea–hypopnoea index (AHI) values were retrieved from the SD card of the CPAP device after six months of therapy. The descriptive statistics of post-treatment AHI values are summarised in Table 7.

Of the 42 patients, 33 (78.6%) had normalised AHI values (<5 events/hour) during CPAP therapy, while 9 (21.4%) had slightly elevated AHI values (>5 events/hour). Importantly, none of the participants—regardless of treatment adherence—had moderate or severe OSA after CPAP treatment, based on the post-treatment AHI scores (Table 8).

Table 9 shows a comparison of pure-tone audiometry results between participants who adhered to CPAP and those who did not. Although some differences were observed at 8000 Hz, no statistically significant differences in hearing thresholds were found between the two groups at any frequency tested.

Table 10 shows transient-evoked otoacoustic emission (TEOAE) results as a function of adherence to CPAP therapy. The signal-to-noise ratio (SNR) values at 2 kHz and 4 kHz were significantly higher in adherent patients compared to non-adherent patients, indicating better function of the cochlear outer hair cell. Although a difference was also observed at 1.42 kHz, it did not reach statistical significance.

After six months of CPAP therapy, otoacoustic distortion-product emission (DPOAE) measurements revealed statistically significant differences in SNR values between adherent and non-adherent participants at frequencies of 1000, 6000, 7000, and 8000 Hz. In all cases, higher SNR values were measured in the adherent group, indicating more favourable cochlear responses. Although differences were found at 1500 Hz and 2000 Hz, these did not reach statistical significance (Table 11).

## 4. Discussion

The two principal pathophysiological mechanisms underlying obstructive sleep apnoea syndrome (OSA) are intermittent hypoxia and sleep fragmentation. Hypoxia plays a central role in the onset and progression of various pathological processes and should not be underestimated. The cardiovascular system, in particular, is adversely affected through pathways involving systemic inflammation, oxidative stress, and heightened adrenergic activity. Both systemic inflammation and oxidative stress stimulate the production of multiple bioactive factors, ultimately leading to endothelial dysfunction—a key contributor to atherogenesis and the development of arterial disease. Oxidative stress promotes atherosclerosis via several mechanisms, including lipid and DNA oxidation, as well as direct impairment of endothelial function. Endothelial dysfunction, in turn, triggers maladaptive vascular responses and is closely linked to the progressive development of atherosclerosis. Furthermore, the combined effects of hypoxia and sleep fragmentation enhance sympathetic nervous system activity, resulting in vasoconstriction and contributing to the development of hypertension. In parallel, sleep fragmentation is associated with increased production of procoagulant factors and impaired hemorheological properties. Together, these mechanisms drive the initiation and progression of various cardiovascular disorders, which in turn further influence the development of other comorbidities associated with obstructive sleep apnoea [16]. Obstructive sleep apnoea is well recognised for its adverse effects on multiple organ systems, including the auditory system; however, the precise nature and mechanisms of cochlear involvement remain incompletely understood [17]. The cochlea is supplied by a single terminal artery and, due to its limited collateral circulation, is highly dependent on adequate oxygenation [18,19]. Recurrent apnoeic episodes can therefore lead to cochlear cell injury. Numerous studies have investigated the pathophysiology of cochlear damage in OSA, with the majority implicating oxidative stress as a primary mechanism underlying hair cell injury [20]. Outer hair cells in the basal turn of the cochlea are particularly vulnerable due to their relatively low activity of glutathione-dependent antioxidant enzymes [20]. While some research has suggested that OSA-related hearing impairment may predominantly result from dysfunction in central auditory pathways [21], most studies indicate that peripheral mechanisms are critical. Chronic intermittent hypoxaemia-induced cochlear ischaemia, combined with inflammation associated with the pro-inflammatory environment of OSA, appears to contribute significantly to hearing impairment [11,22]. For example, Casale et al. reported elevated air-conduction thresholds in OSA patients compared with controls, along with significantly higher SNR TEOAE values [23]. Kayabasi et al. observed that moderate OSA primarily affected high-frequency hearing, whereas severe OSA impaired hearing across all frequencies [17]. Matsumura et al. [24] demonstrated that inner ear damage from severe OSA manifested as reduced DPOAE amplitudes. Similarly, Li et al. reported a significant reduction in DPOAE amplitudes in OSA patients, even in the absence of measurable hearing loss. These findings suggest that chronic cochlear hypoxia in OSA may damage outer hair cells, with changes in DPOAE amplitude preceding detectable alterations in hearing thresholds [25].

This article reports findings from a subset of a larger investigation in which patients with newly diagnosed OSA were compared with healthy controls matched for sex, age, BMI, and comorbidities. This comparison was conducted to demonstrate auditory impairment associated with OSA. These results have been published in the study by Grebenar Cerkez M. et al. Audiologic profile of OSA patients: the effect of chronic nocturnal intermittent hypoxia on auditory function; A Pilot Study, accepted for publication in Acta Clinica Croatica (2024) [12].

The present study investigated the effect of continuous positive airway pressure therapy on auditory function and cochlear outer hair cell activity in patients diagnosed with moderate to severe obstructive sleep apnoea. The main focus was to evaluate the changes in pure-tone audiometry and otoacoustic emissions after six months of CPAP treatment, with emphasis on the role of patient compliance.

In this study, no improvement in hearing thresholds at 500, 1000, 2000, 4000, or 8000 Hz was observed in patients with obstructive sleep apnoea after six months of CPAP therapy. The results therefore do not speak in favour of positive effect of CPAP therapy on hearing thresholds. The signal-to-noise ratio (SNR) values determined for both transient-evoked otoacoustic emissions and distortion-product otoacoustic emissions also showed no statistically significant differences at any of the frequencies analysed.

A review of the available literature revealed a limited number of studies investigating the effect of OSA and its treatment on auditory function, emphasising the novelty and importance of this research. Of the few published papers, most report findings consistent with those presented here. Chi et al. investigated hearing thresholds at low (250 Hz), medium (500–2000 Hz), and high (4000–8000 Hz) frequencies in 28 participants, analysing only one ear per person. They observed an increase in thresholds at medium frequencies after treatment, suggesting possible deterioration rather than improvement [2].

In contrast, a study by Cheung et al. showed no significant effect of CPAP therapy on hearing thresholds [26]. Similar results were reported by Deniz, who found that the use of CPAP did not affect hearing function in either a positive or negative direction [27]. A comprehensive meta-analysis by Kasemsuk et al., which included 20 studies with a total of 34,442 participants, was also unable to demonstrate any significant benefit of CPAP therapy for hearing [28]. Similarly, Mastino et al. concluded in a literature review from 2023 that there was no clear association between CPAP treatment and hearing outcomes [29]. Among other objectives, Faber and colleagues examined the effect of CPAP therapy on hearing improvement after 3 and 6 months of treatment. They used auditory evoked potentials and long-latency responses as their methods of choice, observing a slightly reduced P2 wave latency; however, this finding was not confirmed in subsequent measurements [30].

From a clinical perspective, almost 79% of patients achieved normalised AHI values (<5 events/hour) recorded on the device’s memory cards after six months of auto-CPAP therapy. Importantly, none of the participants had moderate or severe OSA at follow-up, regardless of adherence to therapy. However, the significantly better cochlear responses observed in the adherent group underscore the physiological importance of consistent CPAP use beyond normalisation of AHI.

One area that remains under-researched in the current literature is the role of treatment adherence in influencing auditory outcomes in patients with OSA. Most published studies appear to assume adequate adherence of participants without directly investigating its potential influence. Therefore, a specific aim of this study was to investigate whether adherence to CPAP therapy influences cochlear receptor cell function. Adherence was defined as the use of CPAP for at least four hours per night for at least 70% of nights [7,8]. Based on this criterion, participants were categorised as “adherent” or “non-adherent”. Of the total sample, 13 individuals (31%) were categorised as non-adherent, while 29 (69%) were classified as adherent. These proportions are consistent with previous reports that 20–40% of patients either discontinue therapy or do not fulfil the adherence criteria. The most commonly cited barriers to adherence include nasal mucosal sensitivity, noise from the device and discomfort when with the mask [8,12,31,32]. When comparing hearing thresholds between the two groups using pure-tone audiometry, no statistically significant differences were found at any frequency. Although a difference was found at 8000 Hz, it did not reach statistical significance. These results suggest that treatment adherence has no significant effect on the hearing thresholds of patients with OSA.

In contrast, the TEOAE results showed significantly higher SNR values at 2 kHz (*p* = 0.033) and 4 kHz (*p* = 0.027) in the adherent group, indicating better cochlear function at these intermediate frequencies. Although increased SNR was also observed at 1.42 kHz, the difference was not statistically significant. The DPOAE results showed higher SNR values in the adherent participants at higher frequencies (6000 Hz → *p* = 0.32, 7000 Hz → *p* < 0.001, and 8000 → Hz *p* = 0.021), and at the intermediate frequency of 1000 Hz (*p* = 0.021). These results suggest a positive correlation between adherence to treatment and inner ear function.

A number of authors have investigated differences in audiological assessments and their respective diagnostic value in the detection of hearing impairment. At present, a range of audiological tests is available for the identification of various types of hearing loss—whether conductive, sensory (cochlear), or neural in origin [33]. Given the heterogeneity of aetiologies and the complexity of auditory pathology, there is increasing recognition of the need to employ objective diagnostic tools capable of identifying hearing impairment before its detection via subjective methods, such as pure-tone audiometry. In a study conducted by Kapoor et al., results from pure-tone audiometry were compared with those obtained from distortion-product otoacoustic emissions (DPOAE) in a cohort of military personnel. Their findings indicated that otoacoustic emission testing offers superior sensitivity in the early detection of cochlear damage resulting from noise exposure [34]. Similar observations were reported by Pawlaczyk-Łuszczyńska et al., who compared pure-tone hearing thresholds between music students exposed to chronic noise and a matched control group. While no statistically significant differences were found in audiometric thresholds, the group of music students exhibited reduced DPOAE amplitudes in comparison to controls [35]. These findings collectively support the notion that cochlear (sensory) damage may be present before it becomes clinically evident through subjective audiometric evaluation. Further evidence is provided by Barbee et al., who presented a comprehensive literature review encompassing fifteen studies—seven involving human participants, seven based on animal models, and one incorporating both. Their synthesis concluded that otoacoustic emissions are capable of revealing subclinical (or “hidden”) sensory hearing loss prior to its detection by means of conventional pure-tone audiometry [36].

Although there are no specific studies in the literature looking at audiological differences as a function of CPAP treatment adherence, numerous studies have shown that consistent CPAP use is critical for improving a range of clinical outcomes, including cognitive function, cardiovascular health and stroke risk [37,38]. Our findings extend this understanding and suggest that treatment adherence may also play a role in maintaining or improving cochlear function in patients with OSA.

These findings may suggest that consistent CPAP therapy could contribute to attenuating cochlear damage in OSA, potentially by improving oxygenation and reducing oxidative stress—mechanisms thought to play a role in the pathogenesis of cochlear injury under hypoxic conditions. The absence of improvement in individuals who did not adhere to therapy may indicate that treatment compliance is an important factor in the preservation of inner ear function.

Obstructive sleep apnoea (OSA) is a disorder that affects multiple organs and organ systems. Over the past few decades, increasing attention has been paid to its impact on the development of cardiovascular and cerebrovascular diseases, metabolic, gastrointestinal, and urinary disorders, as well as its effects on cognitive function. In their study, Sircu et al. highlighted the role of OSA in the aetiopathogenesis of various diseases and demonstrated the beneficial effects of continuous positive airway pressure (CPAP) therapy in affected patients. Early diagnosis, together with timely and appropriate treatment, may reduce the incidence of OSA-related comorbidities and decrease the risk of disease-related complications.

Limitations of the study include the relatively short follow-up period of six months and the limited sample size, particularly in the non-adherent group. Although the lack of a healthy control group limits causal interpretation, the within-subject design minimises interindividual variability and enhances sensitivity to treatment-related effects. Future studies should aim to include longitudinal observation of untreated mild OSA cases or delayed-treatment cohorts, as ethically acceptable, and expand to multicentre settings to reinforce causal inference.

## 5. Conclusions

This study found no significant improvement in hearing thresholds after six months of CPAP therapy in patients with moderate to severe obstructive sleep apnoea. However, the otoacoustic emission findings showed that consistent adherence to CPAP treatment was associated with better function of the outer hair cells of the cochlea, particularly at mid and high frequencies. These results suggest that while CPAP does not measurably improve pure-tone hearing, it may play a protective role in maintaining the integrity of the cochlea, likely by improving oxygenation and reducing oxidative stress.

Given the limited number of studies examining hearing in the context of CPAP fidelity, these findings provide new insights into the potential physiological benefits of consistent therapy use. The lack of functional improvement in patients who do not adhere to therapy underscores the importance of compliance in achieving therapeutic outcomes beyond normalisation of breathing. Our results emphasise the importance of promoting compliance with CPAP therapy not only for cardiovascular and neurological protection, but also for the maintenance of auditory health.

Future studies with larger cohorts and longer follow-up are needed to validate these findings and better define the long-term audiological effects of CPAP therapy in this population.

## Figures and Tables

**Table 1:** Descriptive statistics of age of OSA patients.

Descriptive Statistic	Age
N	42
Mean (SD)	52.43 (10.73)
Range	29.00–69.00

Legend: N = Number; SD = Standard deviation: OSA = Obstructive sleep apnoea.

**Table 2:** Distribution of patients according to the degree of apnoea.

Variable		OSA Patients
AHI	Moderate OSA (15–30) Severe OSA (˃30)	11 (26.2%) 31 (73.8%)
	Total	42 (100%)

Legend: OSA = Obstructive sleep apnoea; AHI = Apnoea-Hypopnoea Index.

**Table 3:** Results of pure-tone audiometry analysis.

Variable	Descriptive Statistics and Results of Testing	OSA Group Before CPAP Therapy	OSA Group After 6 Months of Therapy
PTA 500 Hz	N	84	84
	Mean (SD)	14.11 (9.02)	13.51 (5.69)
	Range	10.00–65.00	10.00–45.00
	*p*-value	*p* = 0.411 *	
PTA 1000 Hz	N	84	84
	Mean (SD)	18.27 (12.16)	18.15 (13.05)
	Range	10.00–85.00	10.00–75.00
	*p*-value	*p* = 0.783 *	
PTA 2000 Hz	N	84	84
	Mean (SD)	19.58 (15.03)	20.3 (15.00)
	Range	10.00–85.00	10.00–80.00
	*p*-value	*p* = 0.228 *	
PTA 4000 Hz	N	84	84
	Mean (SD)	31.96 (19.99)	20.3 (15.00)
	Range	10.00–75.00	10.00–80.00
	*p*-value	*p* = 0.106 *	
PTA 8000 Hz	N	84	84
	Mean (SD)	36.90 (23.40)	37.92 (23.51)
	Range	10.00–105.00	10.00–110.00
	*p*-value	*p* = 0.211 *	

Legend: PTA = pure-tone audiometry; N = Number; SD = Standard deviation; OSA = Obstructive sleep apnoea; * *t*-test.

**Table 4:** Results of TEOAE analysis.

Variable	Descriptive Statistics and Results of Testing	OSA Group Before CPAP Therapy	OSA Group After 6 Months of Therapy
SNR 1.00 kHz	N	84	84
	Mean (SD)	10.11 (9.42)	10.31 (8.53)
	Range	−17.30–36.20	−16.50–26.40
	*p*-value	*p* = 0.826 *	
SNR 1.42 kHz	N	84	84
	Mean (SD)	11.89 (9.04)	12.11 (8.27)
	Range	−15.00–26.60	−14.50–26.60
	*p*-value	*p* = 0.791 *	
SNR 2.00 kHz	N	84	84
	Mean (SD)	8.43 (8.81)	9.46 (8.18)
	Range	−15.00–23.30	−12.20–26.40
	*p*-value	*p* = 0.112 *	
SNR 2.83 kHz	N	84	84
	Mean (SD)	5.20 (8.76)	6 (7.69)
	Range	−12.90–24.80	−17.20–23.10
	*p*-value	*p* = 0.168 *	
SNR 4.00 Hz	N	84	84
	Mean (SD)	2.35 (7.86)	2.93 (6.55)
	Range	−14.50–22.40	−10.00–21.50
	*p*-value	*p* = 0.356 *	

Legend: SNR = signal to noise radio; N = Number; SD = Standard deviation; OSA = Obstructive sleep apnoea; * *t*-test.

**Table 5:** Results of DPOAE analysis.

Variable	Descriptive Statistics and Results of Testing	OSA Group Before CPAP Therapy	OSA Group After 6 Months of Therapy
SNR 500 Hz	N	84	84
	Mean (SD)	0.87 (6.34)	1.69 (5.66)
	Range	−20.2–11.40	−11.00–12.40
	*p*-value	*p* = 0.225 *	
SNR 1000 Hz	N	84	84
	Mean (SD)	6.00 (7.45)	6.24 (5.66)
	Range	−15.00–22.40	−11.00–12.40
	*p*-value	*p* = 0.741 *	
SNR 1500 Hz	N	84	84
	Mean (SD)	9.36 (7.29)	8.90 (7.13)
	Range	−8.10–26.20	−10.20–21.20
	*p*-value	*p* = 0.555 *	
SNR 2000 Hz	N	84	84
	Mean (SD)	8.64 (7.99)	9.05 (7.37)
	Range	−18.10–32.60	−12.70–28.40
	*p*-value	*p* = 0.413 *	
SNR 3000 Hz	N	84	84
	Mean (SD)	8.14 (7.73)	8.62 (7.33)
	Range	−18.80–22.40	−12.10–24.50
	*p*-value	*p* = 0.433 *	
SNR 4000 Hz	N	84	84
	Mean (SD)	8.65 (9.42)	8.86 (7.82)
	Range	−17.60–27.30	−16.00–27.70
	*p*-value	*p* = 0.752 *	
SNR 5000 Hz	N	84	84
	Mean (SD)	7.60 (9.42)	7.72 (9.01)
	Range	−17.80–29.30	−16.60–23.60
	*p*-value	*p* = 0.878 *	
SNR 6000 Hz	N	84	84
	Mean (SD)	4.99 (9.72)	5.62 (9.22)
	Range	−14.10–24.10	−16.60–23.60
	*p*-value	*p* = 0.400 *	
SNR 7000 Hz	N	84	84
	Mean (SD)	3.04 (8.31)	3.25 (7.47)
	Range	−18.10–20.30	−16.60–23.60
	*p*-value	*p* = 0.781 *	
SNR 8000 Hz	N	84	84
	Mean (SD)	2.21 (7.21)	1.96 (6.94)
	Range	−23.20–14.30	−16.00–15.30
	*p*-value	*p* = 0.695 *	

Legend: SNR = signal to noise radio; N = Number; SD = Standard deviation; OSA = Obstructive sleep apnoea; * *t*-test.

**Table 6:** Distribution of target OSA group patients by CPAP therapy adherence.

Adherence	N (%)
No	13 (31.0%)
Yes	29 (69.0%)
Total	42 (100.0%)

Legend: N = Number.

**Table 7:** Descriptive statistics of post-treatment AHI values in the OSA group.

Variable	Value
N	42
Mean (SD)	2.95 (2.71)
Range	0.50–12.20

Legend: N = Number; SD = Standard deviation.

**Table 8:** Distribution of OSA group patients by post-treatment AHI severity.

AHI Category	N (%)
Normal (<5)	33 (78.6%)
Mild OSA (>5)	9 (21.4%)
Total	42 (100.0%)

Legend: AHI = apnoea/hypopnoea index; N = number.

**Table 9:** Results of pure-tone audiometry analysis depending on cooperation.

Variable	Descriptive Statistics and Results of Testing	Non-Adherent	Adherent
PTA 500 Hz	N	26	58
	Mean (SD)	14.81 (7.94)	12.93 (4.30)
	Range	10.00–45.00	10.00–15.00
	*p*-value	*p* = 0.441 *	
PTA 1000 Hz	N	26	58
	Mean (SD)	22.31 (17.22)	16.29 (10.33)
	Range	10.00–75.00	10.00–55.00
	*p*-value	*p* = 0.066 *	
PTA 2000 Hz	N	26	58
	Mean (SD)	24.42 (17.22)	16.29 (10.33)
	Range	10.00–75.00	10.00–55.00
	*p*-value	*p* = 0.134 *	
PTA 4000 Hz	N	26	58
	Mean (SD)	40.00 (24.86)	30.86 (20.76)
	Range	10.00–90.00	10.00–90.00
	*p*-value	*p* = 0.099 *	
PTA 8000 Hz	N	26	58
	Mean (SD)	45.38 (27.27)	34.57 (21.01)
	Range	10.00–110.00	10.00–85.00
	*p*-value	*p* = 0.051 **	

Legend: PTA = pure-tone audiometry; N = Number; SD = Standard deviation; * *t*-test; ** Mann–Whitney test.

**Table 10:** Results of TEOAE analysis depending on cooperation.

Variable	Descriptive Statistics and Results of Testing	Non-Adherent	Adherent
SNR 1.00 kHz	N	26	58
	Mean (SD)	8–35 (9.64)	11.19 (7.91)
	Range	−14.90–26.40	−16.50–26.40
	*p*-value	*p* = 0.160 *	
SNR 1.42 kHz	N	26	58
	Mean (SD)	9.54 (10.01)	13.26 (7.17)
	Range	−12.40–26.60	−14.50–23.80
	*p*-value	*p* = 0.057 *	
**SNR 2.00 kHz**	N	26	58
	Mean (SD)	6.64 (8.14)	10.73 (7.95)
	Range	−15.00–23.30	−9.50–26.40
	*p*-value	***p* = 0.033** *	
SNR 2.83 kHz	N	26	58
	Mean (SD)	4.24 (7.28)	6.80 (7.80)
	Range	−11.10–14.30	−17.20–23.10
	*p*-value	*p* = 0.168 *	
**SNR 4.00 Hz**	N	26	58
	Mean (SD)	0.59 (5.99)	3.98 (6.56)
	Range	−10.00–11.10	−10.00–21.50
	*p*-value	***p*** = 0.027 *	

Legend: SNR = signal to Noise ratio; N = Number; SD = Standard deviation; the most important results are marked with bold; * *t* test.

**Table 11:** Results of DPOAE analysis depending on cooperation.

Variable	Descriptive Statistics and Results of Testing	Non-Adherent	Adherent
SNR 500 Hz	N	26	58
	Mean (SD)	0.46 (5.42)	2.24 (5.72)
	Range	−8.80–11.60	−11.00–12.40
	*p*-value	*p* = 0.184 *	
**SNR 1000 Hz**	N	26	58
	Mean (SD)	2.83 (9.76)	7.77 (5.39)
	Range	−13.80–23.70	−5.90–18.80
	*p*-value	***p*** = 0.021 *	
SNR 1500 Hz	N	26	58
	Mean (SD)	6.75 (7.76)	9.86 (6.67)
	Range	−10.20–21.20	−10.20–20.90
	*p*-value	*p* = 0.065 *	
SNR 2000 Hz	N	26	
	Mean (SD)	6.80 (7.12)	58
	Range	−6.90–19.50	10.06 (7.32)
	*p*-value	*p* = 0.061 *	−12.70–28.40
SNR 3000 Hz	N	26	58
	Mean (SD)	7.81 (6.65)	8.62 (7.33)
	Range	−6.00–18.30	−12.10–24.50
	*p*-value	*p* = 0.500 *	
SNR 4000 Hz	N	26	58
	Mean (SD)	6.43 (7.66)	9.96 (7.70)
	Range	−16.00–19.10	−8.00–25.70
	*p*-value	*p* = 0.055 *	
SNR 5000 Hz	N	26	58
	Mean (SD)	5.98 (9.03)	8.50 (8.97)
	Range	−7.60–23.40	−7.50–27.50
	*p*-value	*p* = 0.238 *	
**SNR 6000 Hz**	N	26	58
	Mean (SD)	2.40 (8.30)	7.06 (9.31)
	Range	−15.00–17.40	−16.60–23.60
	*p*-value	***p*** = 0.032 *	
**SNR 7000 Hz**	N	26	58
	Mean (SD)	−1.21 (6.50)	5.24 (7.05)
	Range	−12.60–11.60	−15.60–17.80
	*p*-value	***p*** < 0.001 *	
**SNR 8000 Hz**	N	26	58
	Mean (SD)	−0.63 (5.17)	3.12 (7.34)
	Range	−9.30–2.80	−16.00–15.30
	*p*-value	***p*** = 0.021 *	

Legend: SNR = signal to Noise ratio; N = Number; SD = Standard deviation; the most important results are marked with bold; * *t*-test.

## Data Availability

The original contributions presented in this study are included in the article. Further inquiries can be directed to the corresponding author.

## Abbreviations

The following abbreviations are used in this manuscript:

OSA	obstructive sleep apnoea
PTA	pure-tone audiometry
TEOAE	transient-evoked otoacoustic emission
DPOAE	distortion-product otoacoustic emission
CPAP	continuous positive airway pressure
AHI	apnoea/hypopnoea index
BiPAP	bi-level positive airway pressure
APAP	auto-titrating positive airway pressure
OAE	otoacoustic emission
SD	secure device
SNR	signal to noise ratio
